# Gold Incorporated Mesoporous Silica Thin Film Model Surface as a Robust SERS and Catalytically Active Substrate

**DOI:** 10.3390/molecules21050667

**Published:** 2016-05-20

**Authors:** Anandakumari Chandrasekharan Sunil Sekhar, Chathakudath Prabhakaran Vinod

**Affiliations:** 1 Catalysis Division, CSIR-National Chemical Laboratory, Dr Homi Bhabha Road, Pune 411 008, India; 2 Academy of Scientific and Innovative Research (AcSIR), Anusandhan Bhawan, 2, Rafi Marg, New Delhi 110 001, India; 3 Center of Excellence on Surface Science, CSIR-National Chemical Laboratory, Dr Homi Bhabha Road, Pune 411 008, India

**Keywords:** thin films, model catalysis, SERS, gold nanoparticles

## Abstract

Ultra-small gold nanoparticles incorporated in mesoporous silica thin films with accessible pore channels perpendicular to the substrate are prepared by a modified sol-gel method. The simple and easy spin coating technique is applied here to make homogeneous thin films. The surface characterization using FESEM shows crack-free films with a perpendicular pore arrangement. The applicability of these thin films as catalysts as well as a robust SERS active substrate for model catalysis study is tested. Compared to bare silica film our gold incorporated silica, GSM-23F gave an enhancement factor of 10^3^ for RhB with a laser source 633 nm. The reduction reaction of *p*-nitrophenol with sodium borohydride from our thin films shows a decrease in peak intensity corresponding to –NO_2_ group as time proceeds, confirming the catalytic activity. Such model surfaces can potentially bridge the material gap between a real catalytic system and surface science studies.

## 1. Introduction

The SERS technique using colloidal nanoparticles (NPs) has emerged as a new arena for chemical analysis and single molecule detection since the end of the last century [1], yet most of the time it is a challenge for the research community to develop reproducible surface enhanced Raman scattering (SERS) substrate retaining its higher enhancement factor (EF). The capability to distinguish between the components in a mixture without their separation gives an upper hand for SERS analysis above other similar spectroscopic techniques though it is associated with a smaller molecular cross-section for analysis [2]. The technique utilizes the surface plasmon resonance of the metal NPs to overcome the weak signals in the conventional Raman spectroscopic measurement hindered by the very low efficiency of inelastic photon scattering by molecules. The colloidal metal nanoparticles are well established SERS materials, but these are often associated with poor result reproducibility. This limitation of SERS substrates hindered the development of Raman scattering as a useful analytical technique for decades. Hence there is a huge scope for the development of easily synthesizable, stable SERS substrates for various applications [3]. There are two widely accepted mechanisms for the SERS signal amplification phenomenon: (a) electromagnetic enhancement and (b) chemical enhancement. The electromagnetic enhancement results in 10^7^–10^10^ Raman signal enhancement as a result of the amplification of the electromagnetic field of the metal by the surface plasmon resonance; larger nanoparticles as well as rough nanostructures are believed to provide enhancement based on this mechanism. In the chemical enhancement mechanism, the analyte is considered as directly bonded to the metal nanoparticles and thus a change in polarizability of the molecule which leads to the amplification of SERS intensity up to 10^2^–10^3^. Most of the time the electromagnetic enhancement mechanism is kept in mind while synthesizing metal nanoparticles for SERS applications. But for catalysis, generally the smaller nanoparticles or the undercoordinated atoms in these nanoparticles catalyze the reactions. Hence synthesis of smaller nanoparticles incorporated in porous thin films can effect catalysis as well as signal enhancement in SERS analysis (based on chemical enhancement mechanism) theoretically, which can sort out the mechanistic aspects of the reaction in one shot. Like SERS and *in situ* Raman analysis, in the last few decades *in situ* techniques [4,5,6] have contributed much to our understanding of the fundamental chemistry behind gold catalysis.

Model systems like oxide films incorporated with metal nanoparticles are considered to be a promising tool for surface science techniques as they avoid many of the complexities associated with bulk powder materials/catalysts [7,8,9,10]. Probing the interface during the reaction by surface science techniques will lead to a deeper insight into the fundamental chemistry behind many of reactions which are not yet clearly revealed. The inherent surface plasmon resonance of metal nanoparticles offers wide application as SERS active substrates in various fields [11,12,13,14]. Among all the metal NPs gold [15,16] occupies a unique place in this field because of its UV-visible absorption, normally falling in the region which is commonly used for Raman spectroscopy. Though gold NP colloids show high signal enhancement in SERS there are unable to give reproducible results in the colloidal form mainly due to the instability of colloidal NPs in solution or due to the masking of the active surface with the capping agents. Therefore fabrication of such colloidal particles on various substrates becomes mandatory for better results as well as for repeated use [17,18]. Solid SERS active surfaces are an efficient alternative for reproducible and reliable SERS results. Mesoporous oxide materials are widely used as catalysts and support materials in catalysis. Their advantages such as high surface area, large pore diameter and pore volume of mesoporous materials makes them ideal systems to entrap metal NPs. Surfactant templated synthesis of thin films are well known to the research communities, but the low surface free energy contribution makes these surfactant micelles orient parallel to the surface of the substrate while forming thin films [19,20]. As a result the pore channels will be oriented parallel to the surface. Therefore it is still a challenge to make mesoporous thin films with pore channels perpendicular to the film surface. Moreover for a catalytic reaction to happen the pore channels should be easily available/accessible for the passage of reactants/products in and out of the system throughout the process. The ease of accessibility of pore channels is necessary to avoid any mass transfer limitations during catalysis. For that reason researchers are always interested in making porous thin films with pores oriented perpendicular to the surface. The simplicity of the sol-gel synthesis method and the spin coating technique makes them a promising route for making homogeneous and flat nanoparticle-incorporating thin films. Many triblock polymers such as P123, F127 *etc.*, have been known as structure-directing agents for mesoporous silica synthesis as well as reducing agents for noble metal nanoparticles for decades, yet the literature speaks much less about the application of these block copolymers as reducing and structure-directing agents within the same system [21,22]. Hence making use of the ability of a structure directing agent as the reducing agent during the synthesis of a composite material can reduce the complexity of the synthesis procedure. Further, gold nanoparticles have a drawback which is their weak affinity towards silica surfaces due to which sintering of the nanoparticles severely hampers the catalyst performance. To prevent this, the silica support is functionalized with moieties like aminopropyltrimethoxysilane (APTMS) for anchoring gold nanoparticles to avoid the sintering and other associated problems [23].

Here we have utilized the dual functionality of the triblock copolymer P123 as reducing agent for the generation of gold nanoparticles and the structure directing agent for mesoporous silica synthesis. In our one pot *in situ* approach the metal nanoparticles are reduced within the host silica matrix by P123 during ageing. The novelty of this synthesis method lies in the fact that a higher gold loading of ~25 wt % is achieved in mesoporous silica matrix by this relatively easy and simple technique. The application of P123 as the structure directing agent as well as the reducing agent (for gold precursors) for the composite thin film synthesis is another novelty. The resulting composite film exhibited homogeneous film characteristics as well as SERS activity. Further, the current synthesis procedure results in the formation of gold nanoparticles of similar dimensions equally separated by amorphous silica walls; hence avoiding the mobility of active nanoparticles during the Raman analysis, which is a common cause of non-reproducibility in colloidal SERS systems. The light scattering ability of the nanoparticles largely depends on their particle size [24,25]; therefore we believe that making ultra-small nanoparticles within a mesoporous material will results in the development of a new set of SERS substrates with improved sensitivity and reliability based on the chemical enhancement mechanism. Moreover, from the catalytic point of view, the small nanoparticles give good reactivity for most reactions, even at higher temperatures, especially in the case of gold. A huge volume of reports are available for the conversion of *p*-nitrothiophenol to *p*-aminothiophenol using Raman spectroscopic techniques as well as use of these chemicals as Raman reporter molecules owing to the inherent adhesion property of the thiol groups for gold nanoparticles [26,27,28,29,30]. *p*-Nitrophenol, another industrially important organic molecule and its conversion into *p*-aminophenol is widely studied using UV-Vis spectroscopic techniques and there are limited literature results in which Raman spectroscopy is used to track this conversion reaction catalyzed by gold nanoparticles [31,32,33]. Hence we tried to focus on this reaction using our thin film (GSM-23F) by using Raman spectroscopy.

It has been previously been reported by Weckhuysen *et al*. that plasmonic nanoparticles can be utilized for better understanding of reaction mechanisms through *in situ* Raman as well as tip- enhanced Raman techniques [34,35]. A composite thin film in this regard matches more with the conventional catalytic systems with high surface specificity required for elucidating the reaction pathways of real world catalysis. The purpose of the present study were: (i) the synthesis of small gold nanoparticle-incorporating mesoporous silica thin films using a simples sol-gel method coupled with the spin coating technique; (ii) testing the applicability of such a substrate for SERS effects; (iii) using the model thin film surface and the surface enhancement effects to probe the reaction pathway by monitoring functional groups using Raman spectroscopy; (iv) correlating the catalytic activity of the ultra-small gold nanoparticles with their enhancement factor in the SERS activity. The development of such thin film surfaces can be utilized for understanding reaction mechanism at high temperature and pressure, especially using Raman spectroscopy thereby bridging the material gap that exists in surface science.

## 2. Results

### Structural and Textural Characterisation

**Figure 1:**
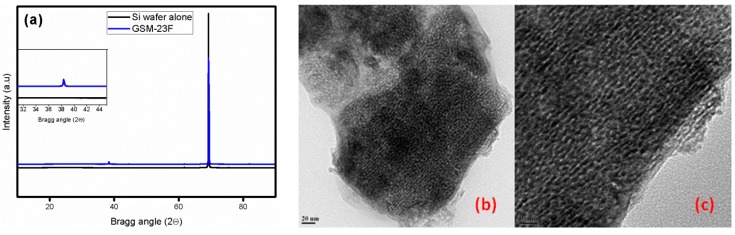
Wide angle XRD spectra of the bare silicon wafer and the GSM-23F film (**a**) TEM analysis of the GSM-23F thin film with different magnifications, scale bars (**b**) 20 nm and (**c**) 10 nm.

Wide angle XRD analysis of the thin film (GSM-23F) gives peaks corresponding to the formation of face centred cubic (fcc) gold nanoparticles within the mesoporous silica support (Figure 1). The periodicity of the mesoporous silica films could not be confirmed by low angle XRD because of the absence of any low angle peaks in the spectrum. Alexandris *et al.* [36] previously reported a detailed study on the reduction mechanism of gold by triblock copolymers such as F127, P123, *etc.*, where they showed the efficient reduction of gold nanoparticles by the PEO surface cavities and uninterrupted nucleation without any structure transition. Additionally Stucky *et al.* [37] elaborated on the applicability of P123 for the synthesis of different silica film mesophases. The applicability of P123 as a reducing agent under our synthesis conditions as well as a structure directing agent for silica synthesis was reported previously [38]. The solid state UV-Vis analysis of the film coated on a FTO plate gave a small broad peak at 510 nm indicating the formation of small gold nanoparticles within the system (Figure S1). The absence of a low angle peak in the small angle X-ray diffraction was reported in the past by Shi *et al.* [39] in a system similar to GSM-23F though their synthesis strategy involved surface modification of silica films. Hence we concluded that the incorporation of gold nanoparticles within the mesochannels could be the reason for the lack of any low angle peak in our case. Further, the intended loading of 25 wt % was achieved according to both the ICP-AES analysis as well as the EDX analysis of the transmission electron microscopy data (Figure S2). The high resolution TEM images in Figure 1b,c clearly show the presence of mesoporosity, yet it is really difficult to distinguish between gold nanoparticles and the empty pores which leads us to believe that small Au particles are trapped inside the channels. The rough pore size estimate from the TEM images is 3.5 nm. The high resolution TEM images prove the success of the synthesis procedure as most of the pores are filled with gold nanoparticles. SAED analysis (not shown here) while doing TEM also failed to give any signature for the lattice parameters for gold; we believe that this discrepancy between XRD and TEM is mainly because of the signal averaging used in XRD analysis. We do observe some bigger gold nanoparticles on the amorphous silica regions in the TEM analysis (see Figure S3 TEM) which are possibly formed due to agglomeration of the particles which are not trapped inside the channels. Interestingly these regions containing gold nanoparticles with sizes ranging from 20 to 60 nm showed no observable porosity on the silica matrix. Robertson *et al.* [40] previously reported that the silica thin films spin cast on gold coated glass slides gave insulating films without any observable pores. Their synthesis did not involve any porogen, whereas in our case we used P123 as the structure directing agent for mesoporous silica synthesis. Therefore P123 along with the ultra-small gold nanoparticles are the reasons for maintaining observable mesoporosity in our case.

**Figure 2:**
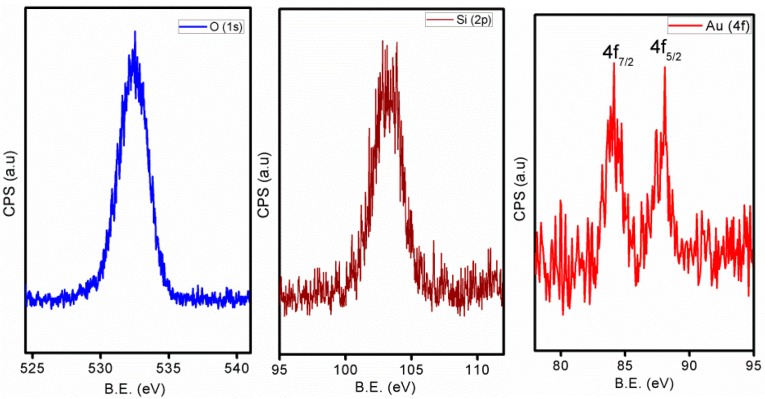
XPS data of the GSM-23F thin film after calcination.

Nitrogen adsorption-desorption of the bulk form of these mesoporous thin films showed a type-IV isotherm (Figure S4). The BET surface area is found to be 545 m^2^·g^−1^ and the pore size to be 3.5 nm. This pore size value is consistent with that obtained from the TEM analysis. To explore the chemical composition of the active nanoparticles we did XPS analysis (Figure 2). The results clearly indicate the existence of gold in the zero valent state with a Au(4f_7/2_) feature ate 84 eV. The O1s and Si(2p) binding energy value are also consistent with the silica values. These metallic gold nanoparticles are known for their catalytic activity as well as SERS enhancement.

**Figure 3:**
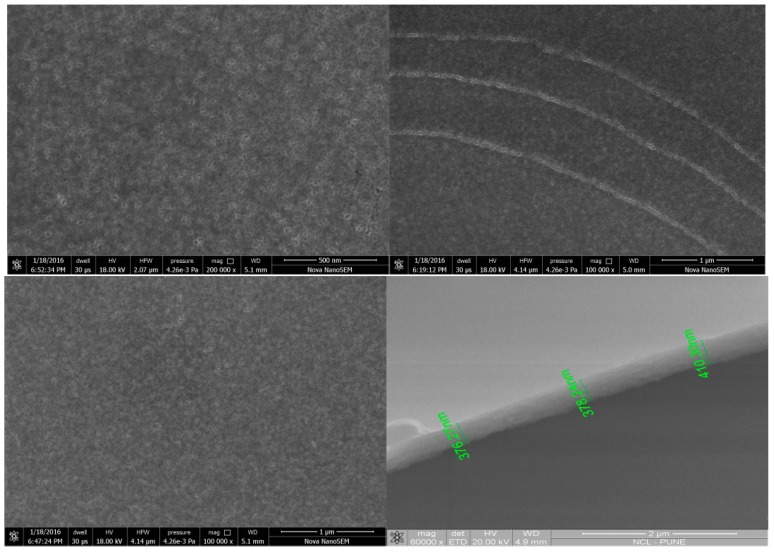
Scanning electron microscopy images of the thin film GSM-23F on various magnifications and the cross-sectional analysis of such a thin film.

In order to understand the three dimensional morphology of the thin films formed on the Si (100) substrate we have carried out FESEM analysis. Formation of crack-free films at the macroscopic level is clearly observable in the low resolution FESEM images. In addition to that accessible pore channels oriented perpendicular to the substrate is evident from the FESEM images at higher resolutions (Figure 3).

**Figure 4:**
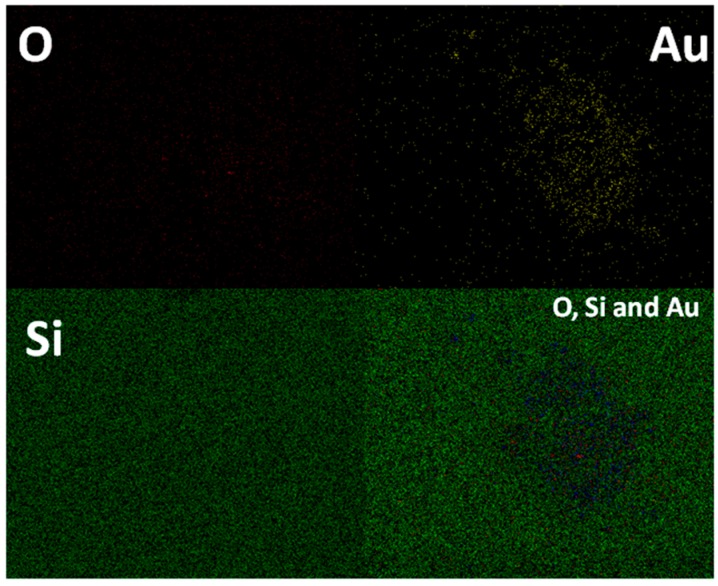
Elemental mapping of the GSM-23F thin film done under scanning electron microscope.

The elemental mapping analysis done on thin film samples further exemplifies the distribution of small gold nanoparticles in a uniform manner on the pore channels (Figure 4). The pore channels which are easily accessible for reactants to reach the active gold nanoparticles make it a live template for catalytic reactions. Moreover the well separated ultra-small gold nanoparticles can generate hot spots under suitable conditions to become an important analytical tool for SERS analysis. The catalytic activity as well as the applicability as a SERS active substrate is further confirmed by tracking the signal enhancement of the dye Rhodamine B (10^−8^ M) and the catalytic reduction of *p*-nitrophenol by micro-Raman analysis.

## 3. Discussion

The properties of an ideal catalytic system in our GSM-23F thin film is evident in all the characterizations mentioned above. The high porosity observed in the TEM and SEM (Figure 1 and Figure 3) directly points to the ease of access of the reactants/products during the catalytic reaction. Similarly, signal amplification can be expected while analysing the SERS of the probe molecule Rhodamine B based on the chemical enhancement mechanism. Making use of a catalytic system for the SERS analysis could potentially result in the development of multi-tasking materials which could sense an analyte in a mixture and easily catalyse the reaction towards the direction of the desired product. A detailed understanding of the SERS and catalytic application of our GSM-23F thin film is discussed in the following section.

### 3.1. GSM-23F as a Robust SERS Substrate

**Figure 5:**
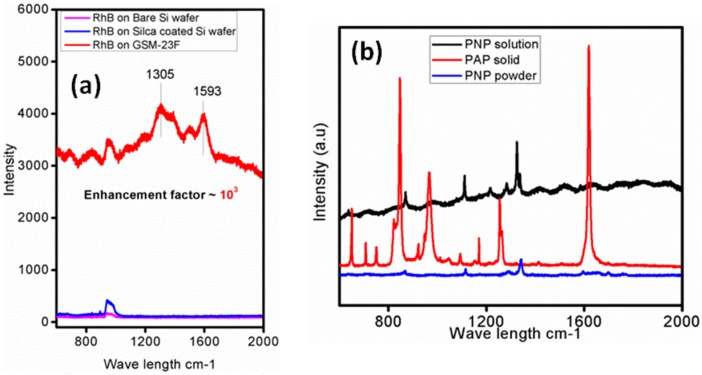
SERS spectra of rhodamine B on GSM-23F thin film and the bare silicon substrate (**a**) and the Raman spectra of the 4-nitrophenol (PNP), 4-aminophenol (PAP) (**b**) under 633 nm laser excitation source.

The incorporation of ultra-small gold nanoparticles within the mesopores of the silica matrix can develop hot spots, which on the application of suitable resonance frequency will act as SERS probes. The large Raman cross-section along with ample number of references in the literature [41,42,43] made us to select Rhodamine B as the probe molecule for the Raman signal enhancement study with our GSM-23F. Therefore any change in the Raman spectrum could be attributed to the interaction of the SERS substrate with the adsorbed probe molecule. As expected our system is able to give an enhancement of 10^3^ with Rhodamine B (10^−8^ M). In the case of gold there are reports saying that as particle size increases the enhancement factor also increases. [42] A large ordered super lattice structures in these *bigger* particles develops strong inter-particle plasmon coupling and thereby a higher SERS enhancement. The ultra-small nanoparticles in GSM-23F produce chemical enhancement in the present study which leads to significant enhancement in the signal intensity for Rhodamine B even at a concentration as low as 10^−8^ M. Further, the synthesised composite thin film was unable to sense the Raman probe molecule, in our case Rhodamine B (RhB) when the concentration was less than 10^−8^ M. Hence we restricted ourselves to not to go beyond this concentration and the enhancement was calculated based on the signal intensity of the probe molecule at 10^−8^ M concentration and compared with that from a bare silicon substrate as well as with silica film-coated silicon substrate. One could barely see any signature of Rhodamine B when a silica film without gold nanoparticles was used as the substrate. Figure 5a clearly express the enhanced intensity, and the enhancement in intensity is calculated by comparing the peak intensity at 1305 cm^−1^ in the silica alone system and GSM-23F. As the gold nanoparticles are well separated by the mesoporous walls of silica the electromagnetic enhancement towards SERS activity cannot be expected. Moreover the lesser affinity of silica towards gold nanoparticles avoids any charge transfer interaction that might result in signal enhancement [44]. Thus, the approximately three orders of magnitude enhancement observed in our case is mainly due to the chemical enhancement theory which explains the relatively smaller enhancement observed here. The enhancement factors were largely reproducible for the composite thin film synthesised from different batches. It is worth mentioning at this point that these materials are primarily developed for catalytic applications and there nanoparticle separation from one another is a rule of thumb for better activity.

### 3.2. GSM-23F As a Catalytically Active Model Surface

**Figure 6:**
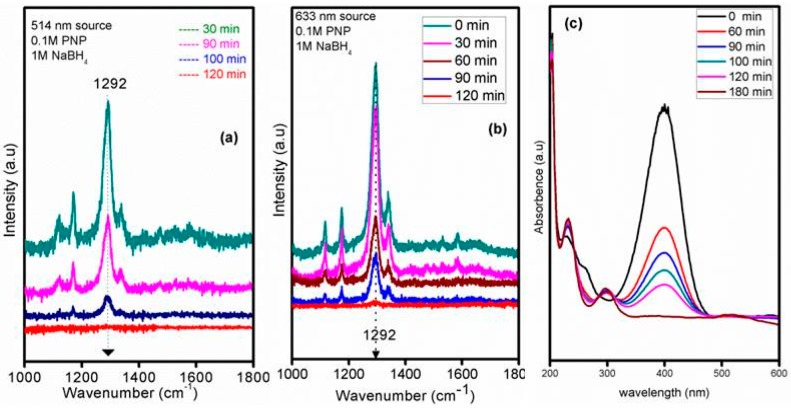
*In situ* Raman spectral data under (**a**) 514 nm (**b**) 633 nm laser source and (**c**) UV-Vis spectral data for the conversion of 4-nitrophenol to 4-aminophenol.

Conversion of aromatic nitro compounds to amino compounds is an important class of reaction in the pharmaceutical and fine chemical industries. Recently Sun *et al.* [45] reported the conversion of *p*-nitrophenol to *p*-aminophenol using silver microflowers and used Raman spectroscopy for *in situ* monitoring of the reaction pathways. There, features corresponding to *p*-nitrophenol were not observed in the Raman spectra. Contrary to that we obtained intense peaks in the spectra of *p*-nitrophenol and could clearly show the decrease in intensity as time progressed and the complete absence of the peak corresponding to –NO_2_ group after the completion of reaction. The lower scattering cross-section of the amino group or the weaker interaction between the gold NPs and *p*-aminophenol could be the reason for the absence of the peak corresponding to the amino group in our Raman spectra. Since our own previous work with a similar system in bulk catalysis proved the existence of ultra-small nanoparticles on these kind of silica matrix, we used a 514 nm laser source for the catalytic activity test first [38]. The literature claims the existence of different vibrational bands in the Raman spectra of PNP as 869 (C-H out of plane bending), 1120 (C-H in plane bending mode) and 1341 cm^−1^ (–NO_2_ asymmetric stretching mode) [46]. In our case the prominent peak positions were 1341 cm^−1^ (for solid 4-nitrophenol), 1324 cm^−1^ (for 4-nitrophenol solution in water) and 1256 and 1621 cm^−1^ (for solid 4-aminophenol) under a 633 nm laser source in the normal Raman spectra (Figure 5b), whereas the prominent peak of the nitro group on the thin film catalysts under the reaction conditions is slightly shifted to 1292 cm^−1^ under the 514 nm laser source and the addition of the reducing agent sodium borohydride brings about the reduction reaction as observed in the intensity of the corresponding nitro group over a period of 100 min and its complete disappearance in 120 min (Figure 6). The Raman analysis profile reflected similar peak positions even when the laser source was changed from 514 nm to 633 nm under our reaction conditions, indicating the versatility of the synthesised thin film catalyst for catalysis as well as Raman studies.

Figure 5b shows the Raman spectra of PNP with wet and dry GSM-23F, where the variation in signal intensity can be attributed to the different orientation of adsorbed molecules on the film surface [47]. To our knowledge there are no reports regarding the Raman study of the reduction of aromatic nitro compounds, specifically *p*-nitrophenol to *p*-aminophenol, using gold nanoparticles incorporated in a support matrix. The absence of any peak in the product stream is understandable as the enhancement obtained was only four times as intense when we took the SERS spectra of *p*-aminophenol in the presence of gold colloids (see Figure S5). In a colloidal system the widely explained SERS mechanism is based on electromagnetic enhancement and in such systems the literature claims an enhancement of the order of 10^4^–10^7^. These results give a stamp of approval to our belief that the low scattering cross-section of *p*-aminophenol is the reason for the lack of peaks in the spectra.

As we were unable to detect the peak corresponding to the –NH_2_ peak in the Raman spectra after the reaction (though the –NO_2_ peak had completely vanished), the formation of PAP is further confirmed by UV-Vis spectra (Figure 6c). The experimental conditions of the UV-Vis and Raman analysis were same; hence there should be no ambiguity in comparing the results. Emergence of a peak at 300 nm with time and the decrease in the peak intensity at 400 nm clearly validate the reduction of PNP with sodium borohydride in the presence of GSM-23F.

Thus the synthesised materials showed promising catalytic activity for the *p*-nitrophenol reduction to *p*-aminophenol which is tracked using Raman spectroscopy as well as UV-Vis spectrophotometry. More studies are ongoing to improve the sensititvity of GSM-23F for amplifying the signal intensity while performing catalysis, so that generation of the product *p*-aminophenol can also be tracked irrespective of its smaller Raman cross-section. The smaller SERS enhancement factor compared to other colloidal or fabricated NP systems is most likely due to the chemical enhancement mechanism. Since the synthesized NPs are ultra-small as well as being separated by porous silica walls, the electromagnetic enhancement mechanism is ruled out in our GSM-23F catalyst.

## 4. Materials and Methods

### 4.1. Thin Film Synthesis

The synthesis of the thin film (hereafter GSM-23F) involves three parts:

(i) Pretreatment of the substrate
(ii) Synthesis of the silica gold sol-gel and sol ageing
(iii) Spin coating, ageing and high temperature calcinations

#### 4.1.1. Pretreatment of the Substrate, Si (100)

The Si (100) wafer 1 × 1 cm^2^ is hydroxylated in boiling water and then calcined at 750 °C for 24 h to generate a native silica layer on the substrate surface. This calcination will avoid any possible formation of silicides or carbides in the final stage. The native silica layer formed substrate is again hydroxylated to generate a better hydrophilicity for the substrate. The Si (100) wafer disc was purchased from Sigma Aldrich (St. Louis, MO, USA) and used after the pretreatment mentioned above.

#### 4.1.2. Silica Gold Sol-Gel Synthesis

A modification of the procedure of Stucky *et al*. [48] was used here. Tetraethyl orthosilicate (TEOS, Aldrich) is used as the silica source and HAuCl_4_·3H_2_O (99.99%; Aldrich) as the precursor for gold nanoparticle synthesis. In a typical synthesis TEOS (1 mL) is added into a mixture of ethanol (8 mL), DI water (3 mL) and HCl (pH = 2, 2 mL) and stirred for half an hour at 50–80 °C. Then 0.1 M HAuCl_4_ (5 mL) is added and the sol is stirred for another 3 h at room temperature. The sol is further kept still at room temperature for another 13 h for ageing. 

#### 4.1.3. Film Casting on the Hydroxylated Si (100)

The 13 hour h aged gold silica sol is then spin coated on a previously calcined and hydroxylated silicon (100) wafer. Spinning speed is maintained at 1200 rpm for 40 s while casting the sol and then at 3200 rpm again for 40 s. The kept at room temperatures for another two days for film ageing followed by calcination of the wafers at 540 °C for 4 h in a muffle furnace with a ramping rate of 2 °C/min.

### 4.2. SERS Experimental

The SERS studies are done using 10^−8^ M aqueous solution of Rhodamine B. Typically, 1 × 1 cm^2^ silicon wafer coated with GSM-23F thin film is completely dipped into 10^−8^ M Rhodamine B (2 mL) for 1 h. The film is then washed with distilled water to remove excess dye from the surface and dried at 60 deg for 1 h prior to Raman analysis using a 633 nm laser source. The same protocol is followed for the bare silicon wafer as well as the silica thin film-coated Si wafer for comparison. For the catalytic reduction of 4-nitrophenol (PNP) to 4-aminophenol (PAP), a 1 × 1 cm^2^ silicon wafer coated with GSM-23F is dipped in a small glass open reactor and sodium borohydride is added in order to start the reaction. Periodically Raman data is collected and analyzed to understand the reaction progress. Typically, 0.1 M PNP (20 μL) is added to a thin film placed in the glass reactor followed by the addition of 0.1 M NaBH_4_ (2.5 mL) and the progress of the reaction is monitored using a Raman instrument equipped with a CCD detector and 514/633 nm laser source. Normal Raman spectra of the standard compounds are analyzed using a 633 nm laser source.

### 4.3. Characterizations

The low and wide angle powder X-ray diffraction (XRD) of all the sample were measured on a PANalytical X’pert Pro dual goniometer diffractometer (Almelo, The Netherlands). A proportional counter detector was used for low angle experiments and an X’celerator solid state detector was employed in wide angle experiments. Cu kα radiation source (1.5418 Å) is used with a Ni filter and data collection was carried out using a flat holder in Bragg-Brentano geometry (0.2°/min). UV-Vis spectra were acquired using Cary 50 Conc UV-Vis spectrophotometer (Agilent, Santa Clara, CA, USA) with a dual beam source. Transmission electron microscopy analysis was measured with Model F20 instrument (Tecnai, Hillsboro, OR, USA) operating at 300 kv. Samples were prepared by mechanically removing (scraping) some part of the film and dispersing it in ethanol with low power sonication before drop casting a sample on a carbon-coated Cu grid for observation. Chemical analysis for Au was carried out in a Spectro Arcos ICP-OES instrument (Kleve, Germany). Standard solutions were used for calibration purpose. Scanning electron microscopy analysis was performed with a model Quanta 200 3D with EDX for elemental analysis (FEI, Hillsboro, OR, USA). A Horiba Jobin Yvon LabRam HR 800 spectrometer (HJY, Kyoto, Japan) was used for Raman analysis with a laser wavelength of 633 nm and 514 nm.

## 5. Conclusions

In conclusion we have reported an easy and convenient way for making gold incorporated porous silica thin films. Characterization revealed that the films are crack-free with perpendicular pore channels. These gold incorporated silica thin films are demonstrated to show SERS properties. Further, the *p*-nitrophenol reduction reaction was used and monitored using Raman spectroscopy as a test reaction and proved the usefulness of the thin film as a catalytically active substrate for model surface science studies. This approach can be used for making other catalytically relevant metal nanoparticle-silica systems for fundamental surface science studies.

## Supplementary Materials

Supplementary materials can be accessed at: http://www.mdpi.com/1420-3049/21/5/667/s1.
