# Supplementary material for: Gold Incorporated Mesoporous Silica Thin Film Model Surface as a Robust SERS and Catalytically Active Substrate
Source: Molecules. 2016 May 20;21(5):667. doi: 10.3390/molecules21050667 (PMC6273419; doi:10.3390/molecules21050667)
Supplement: Supplementary file 1 [file molecules-21-00667-s001.pdf]

Supplementary Materials: Gold Incorporated Mesoporous Silica Thin Film Model Surface as a Robust SERS and Catalytically Active Substrate

Anandakumari Chandrasekharan Sunil Sekhar and Chathakudath Prabhakaran Vinod

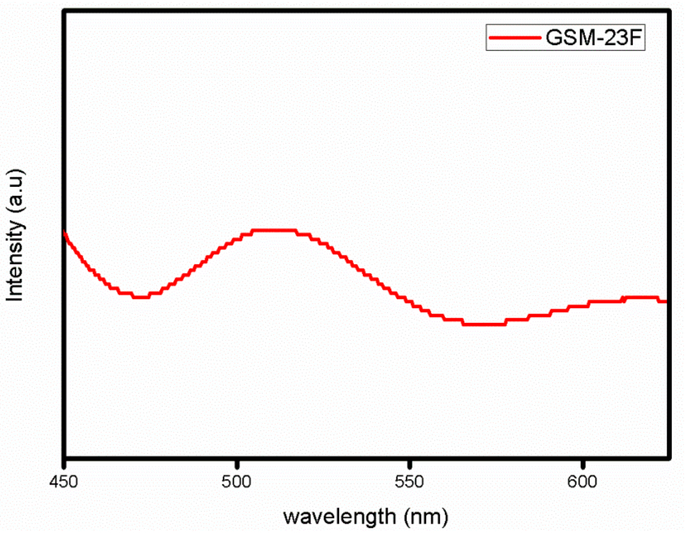

Figure S1. Solid state UV-Vis spectra of the GSM-23F thin film synthesized on an FTO plate.

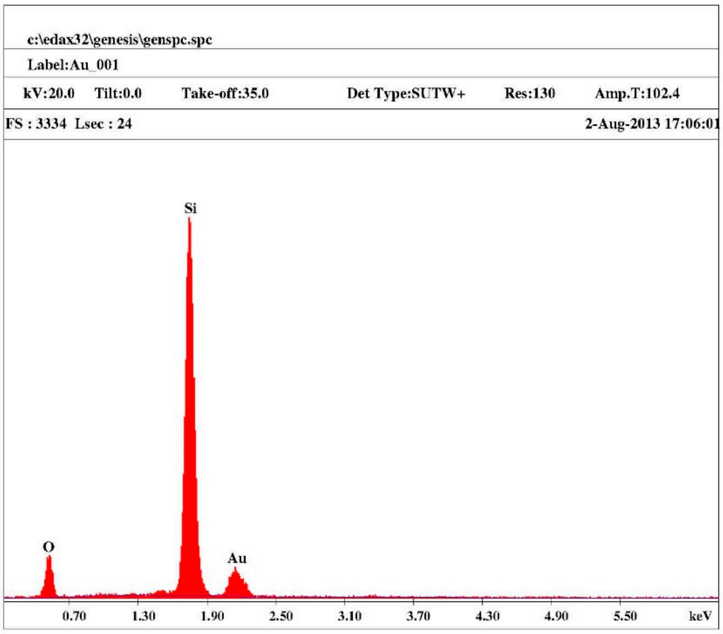

Element	Wt %	At %	K-Ratio	Z	A	F
O K	20.50	38.02	0.0497	1.0808	0.2241	1.0005
SiK	55.21	58.32	0.4619	1.0344	0.8088	1.0000
AuM	24.29	3.66	0.1381	0.7377	0.7705	1.0000
Total	100.00	100.00				

Element	Net Inte.	Bkgd Inte.	Inte. Error	P/B
O K	64.28	1.79	2.58	35.91
SiK	800.10	7.32	0.72	109.26
AuM	65.62	6.71	2.73	9.78

Figure S2. EDX analysis data of the GSM-23F thin film showing the loading of approximately 25 wt % of gold in our thin films which matches well with the ICP-AES analysis.

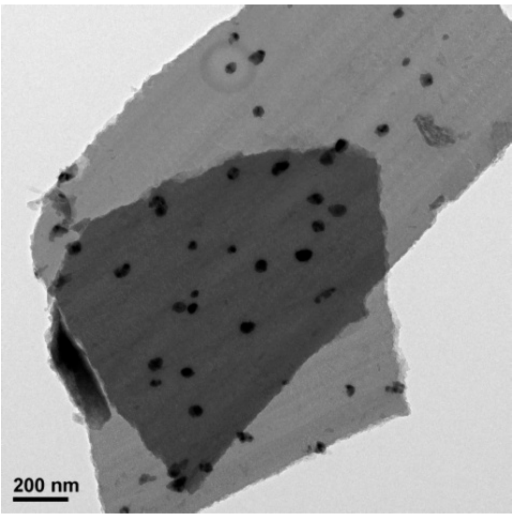

Figure S3. Transmission Electron microscopy image of the GSM-23F thin film showing completely flat film surface with larger gold crystallites.

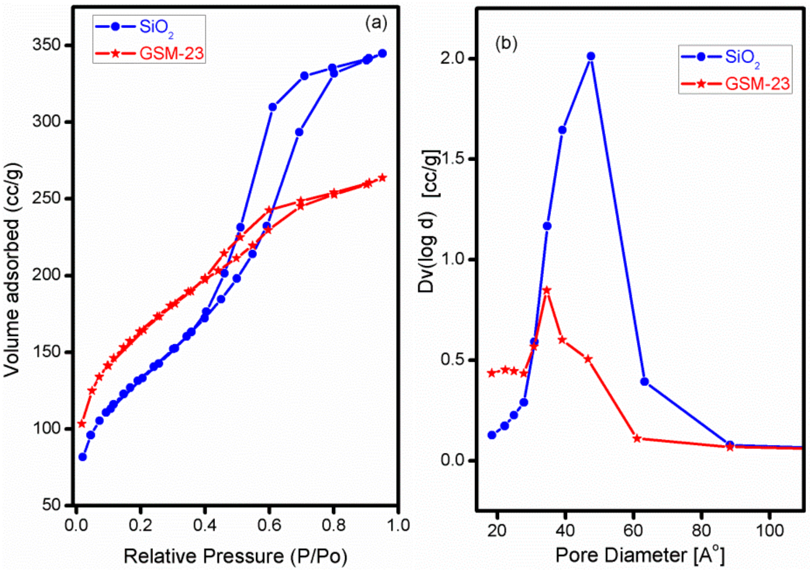

Figure S4. Surface area (**a**) and pore size distribution (**b**) of the bulk counterparts of mesoporous silica and the GSM-23 [1].

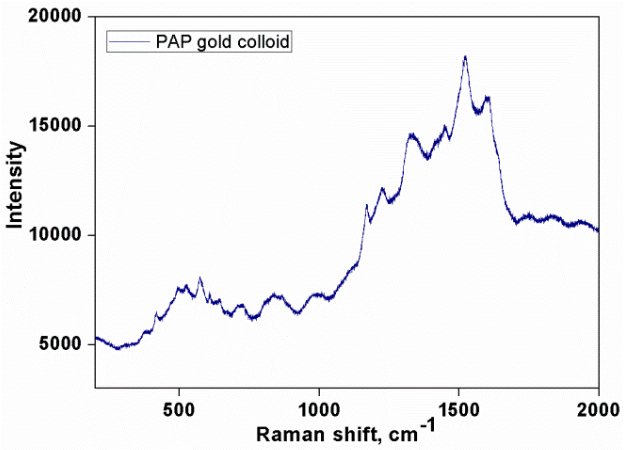

Figure S5. Raman spectral data of 4-aminophenol with Au nanoparticle colloid under 633 nm laser source.

Reference

1. Sunil Sekhar, A.C.; Sivaranjani, K.; Gopinath, C.S.; Vinod, C.P. A simple one pot synthesis of nano gold–mesoporous silica and its oxidation catalysis. *Catal. Today* **2012**, *198*, 92–97.
